# Aspirin Modulation of the Colorectal Cancer-Associated Microbe Fusobacterium nucleatum

**DOI:** 10.1128/mBio.00547-21

**Published:** 2021-04-06

**Authors:** Caitlin A. Brennan, Geicho Nakatsu, Carey Ann Gallini Comeau, David A. Drew, Jonathan N. Glickman, Robert E. Schoen, Andrew T. Chan, Wendy S. Garrett

**Affiliations:** a Department of Immunology and Infectious Diseases, Harvard T. H. Chan School of Public Health, Boston, Massachusetts, USA; b Harvard T. H. Chan Microbiome in Public Health Center, Boston, Massachusetts, USA; c Clinical and Translational Epidemiology Unit, Department of Medicine, Massachusetts General Hospital and Harvard Medical School, Boston, Massachusetts, USA; d Division of Gastroenterology, Department of Medicine, Massachusetts General Hospital and Harvard Medical School, Boston, Massachusetts, USA; e Department of Pathology, Harvard Medical School, Boston, Massachusetts, USA; f Beth Israel Deaconess Medical Center, Boston, Massachusetts, USA; g Division of Gastroenterology, Hepatology, and Nutrition, Department of Medicine, University of Pittsburgh, Pittsburgh, Pennsylvania, USA; h Broad Institute of Harvard and MIT, Cambridge, Massachusetts, USA; i Department and Division of Medical Oncology, Dana-Farber Cancer Institute and Harvard Medical School, Boston, Massachusetts, USA; j Department of Molecular Metabolism, Harvard T. H. Chan School of Public Health, Boston, Massachusetts, USA; Rutgers University

**Keywords:** *Fusobacterium nucleatum*, aspirin, colon cancer

## Abstract

Aspirin is a chemopreventive agent for colorectal adenoma and cancer (CRC) that, like many drugs inclusive of chemotherapeutics, has been investigated for its effects on bacterial growth and virulence gene expression. Given the evolving recognition of the roles for bacteria in CRC, in this work, we investigate the effects of aspirin with a focus on one oncomicrobe—Fusobacterium nucleatum. We show that aspirin and its primary metabolite salicylic acid alter F. nucleatum strain Fn7-1 growth in culture and that aspirin can effectively kill both actively growing and stationary Fn7-1. We also demonstrate that, at levels that do not inhibit growth, aspirin influences Fn7-1 gene expression. To assess whether aspirin modulation of F. nucleatum may be relevant *in vivo*, we use the *Apc^Min/+^* mouse intestinal tumor model in which Fn7-1 is orally inoculated daily to reveal that aspirin-supplemented chow is sufficient to inhibit F. nucleatum-potentiated colonic tumorigenesis. We expand our characterization of aspirin sensitivity across other F. nucleatum strains, including those isolated from human CRC tissues, as well as other CRC-associated microbes, enterotoxigenic Bacteroides fragilis, and colibactin-producing Escherichia coli. Finally, we determine that individuals who use aspirin daily have lower fusobacterial abundance in colon adenoma tissues, as determined by quantitative PCR performed on adenoma DNA. Together, our data support that aspirin has direct antibiotic activity against F. nucleatum strains and suggest that consideration of the potential effects of aspirin on the microbiome holds promise in optimizing risk-benefit assessments for use of aspirin in CRC prevention and management.

## Abstract (precis)

There is an increasing understanding of the clinical correlations and potential mechanistic roles of specific members of the gut and tumoral microbiota in colorectal cancer (CRC) initiation, progression, and survival. However, we have yet to parlay this knowledge into better CRC outcomes through microbially informed diagnostic, preventive, or therapeutic approaches.

## INTRODUCTION

The microbiome and specific members thereof are increasingly recognized for their potential contributions to the initiation and progression of colorectal cancer (CRC). CRC-associated microbes—including Fusobacterium nucleatum, enterotoxigenic Bacteroides fragilis, and colibactin-producing Escherichia coli—influence carcinogenesis through a number of mechanisms, such as: inducing host cell DNA damage (1), shaping the tumor-immune microenvironment (2–4), and promoting metastasis (5, 6). Thus, these bacteria are an increasing focus for CRC diagnostics and therapeutics.

F. nucleatum is a Gram-negative anaerobe found commonly in the human oral cavity but rarely in stool. F. nucleatum is specifically enriched in colonic adenomas and CRC compared with normal colonic tissues (3, 7–11). In cell culture and animal models, F. nucleatum increases intestinal cancer cell proliferation (12, 13), localizes to CRC tissues (14), and influences immune responses within the tumor microenvironment (3, 4). Intratumoral fusobacterial burden is associated with poorer patient prognosis (15, 16) and CRC recurrence after treatment (17). These observations support the idea that F. nucleatum is a potential target for CRC prevention and treatment. A preclinical study using metronidazole demonstrated slower growth of mouse-implanted patient-derived xenografts harboring F. nucleatum (18), providing a proof of concept that modulating F. nucleatum levels could slow CRC growth. However, antibiotic resistance and antibiotic-induced dysbiosis highlight the need to identify alternative agents that might similarly be used without such concerns.

Aspirin, acetylsalicylic acid, is a nonsteroidal anti-inflammatory drug (NSAID) that targets cyclooxygenase-2 (COX-2; or prostaglandin-endoperoxide synthase 2) to inhibit prostaglandin biosynthesis (19, 20). Widely used for pain and inflammation, aspirin is recommended by the United States Preventive Services Task Force to prevent CRC and cardiovascular disease in certain populations (21). Meta-analyses support aspirin as a highly effective CRC chemopreventive treatment (22, 23). However, conflicting results in mouse models (24–26), as well as a recent work in which concurrent antibiotic treatment was necessary to observe aspirin’s antitumoral effects (27), suggest that part of aspirin’s efficacy as a CRC chemopreventive may be mediated by the microbiota. Aspirin and other NSAIDs cause shifts in the microbiota (28–30). Aspirin and salicylic acid, its primary bioactive metabolite, directly affect bacteria by inhibiting growth (31, 32) and altering virulence factor expression (33–37). While some bacteria have specific salicylic acid-responsive regulators (38, 39), how aspirin and salicylic acid drive these changes in other bacteria is less understood. Given these responses, the microbiota has been proposed as a potential mechanism for aspirin chemoprevention and a rich target for precision prevention biomarkers (40).

As aspirin is already employed for CRC prevention, although it is underutilized, and can influence other bacteria through both its antimicrobial and regulatory effects, we posited that aspirin might affect F. nucleatum growth or behavior. Here, we examine the effects of aspirin on F. nucleatum both in culture and during tumorigenesis to ascertain if aspirin holds potential for modulating F. nucleatum-associated CRC outcomes.

## RESULTS

### Aspirin and salicylic acid alter Fn7-1 growth in culture.

**FIG 1:**
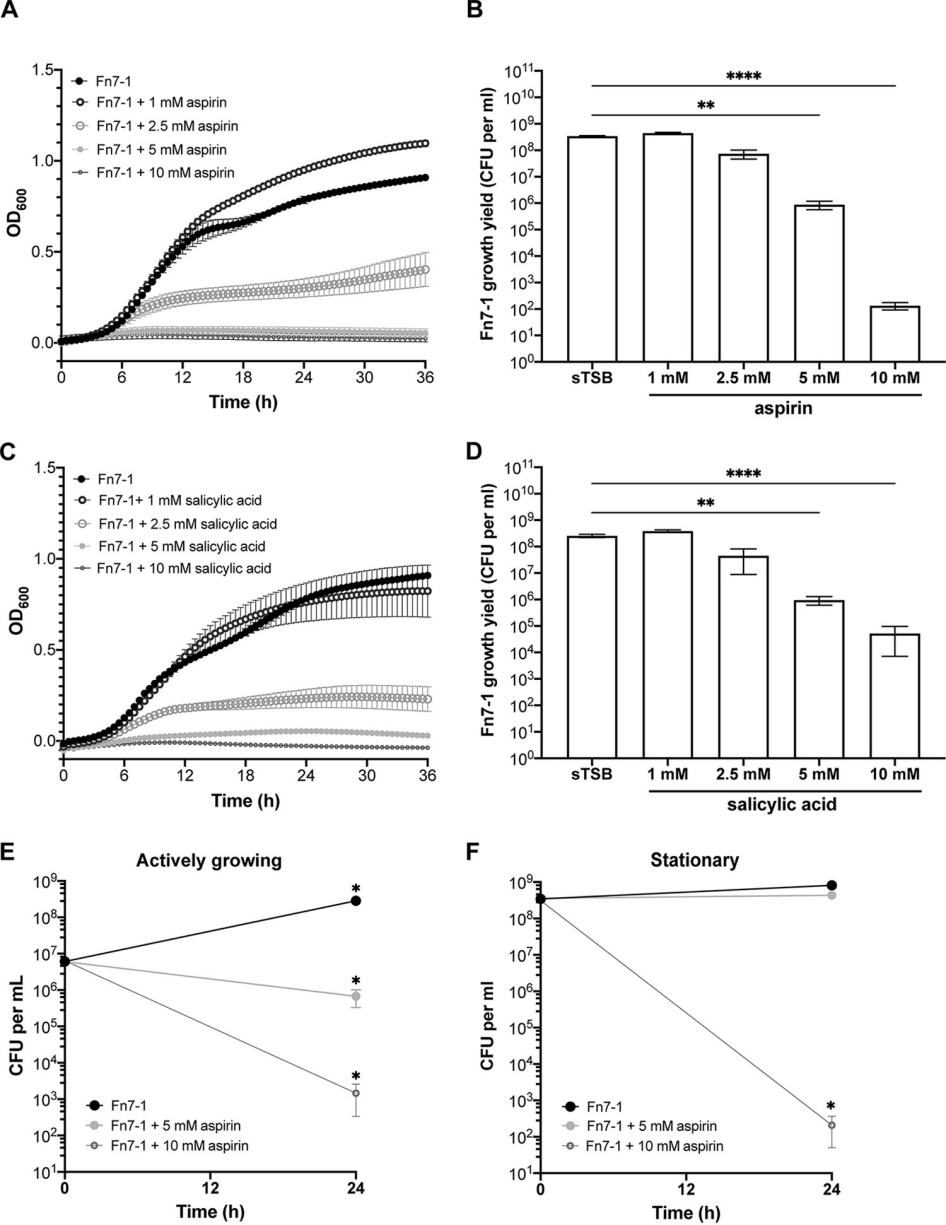
Fn7-1 responses to aspirin and salicylic acid in culture. (A and C) Growth curve as determined by OD_600_ for Fn7-1 grown in sTSB supplemented with different concentrations of aspirin or salicylic acid. (B and D) Fn7-1 growth after 24 h in indicated media, as determined by final CFUs per ml of culture. (E and F) Fn7-1 growth at t_0_ and after 24 h in indicated media, as determined by CFU per ml of culture. For actively growing cells (E), cultures were inoculated 1:100 from an overnight preculture into indicated media. For stationary-phase cells (F), cells were pelleted and washed into fresh, prereduced indicated media. All data represent the mean ± SEM for at least 6 cultures. For A and C, data were analyzed by two-way repeated measures analysis of variance (ANOVA) with *post hoc* Dunnett’s test. For aspirin, samples are significantly different from sTSB at *P* values of <0.05 beginning at 7.5 h (5 mM and 10 mM), 9.5 h (2.5 mM), or 17 h (1 mM). For salicylic acid, samples are significantly different from sTSB at *P* values of <0.05 beginning at 8 h (10 mM), 8.5 h (5 mM), or 11.5 h (2.5 mM). For B and D, ** indicates *P* values of <0.01 and **** indicates *P* values of <0.0001 as determined by Kruskal-Wallis test with *post hoc* Dunn’s test for multiple comparisons to the control medium sample. For E and F, Wilcoxon signed-rank test was performed for each condition at 24 h compared with t_0_. *, *P* < 0.05.

To probe whether aspirin affects the well-studied F. nucleatum strain Fn7-1, we assayed growth in media supplemented with increasing aspirin concentrations comparable to those used in prior studies (31, 33, 41) (Fig. 1A). We adjusted the pH of all media after aspirin or salicylic acid addition to match the control medium, to avoid conflating aspirin-specific and pH-based responses. In 1 mM aspirin, Fn7-1 growth mildly increased, as determined by maximum optical density (optical density at 600 nm [OD_600_]), despite indistinguishable log-phase growth from Fn7-1 grown in control medium (supplemented tryptic soy broth [sTSB]) (Fig. 1A). However, higher levels of aspirin slowed or entirely inhibited Fn7-1 growth. Because OD_600_ is a proxy for bacterial abundance and affected by aggregation and cell morphology, we determined growth yield by CFUs from cultures grown in the presence or absence of aspirin for 24 h (Fig. 1B). Beginning at 2.5 mM aspirin, we observed decreased growth yield (4-fold, but not statistically significant) compared with that in sTSB. In 5 mM and 10 mM aspirin, Fn7-1 growth yield was significantly reduced by 388-fold and over 10^6^-fold, respectively. These data demonstrate that aspirin can inhibit Fn7-1 growth in culture.

We next probed whether salicylic acid, aspirin’s primary metabolite, affected Fn7-1 growth. In OD_600_ and growth yield experiments (Fig. 1C and D), we observed similar inhibition of Fn7-1 growth. While there are subtle differences between the responses to aspirin and salicylic acid, such as greater sensitivity to 10 mM aspirin than to 10 mM salicylic acid, the responses to both compounds were largely similar. Because of the clinical relevance of aspirin in contrast to salicylic acid, we focused on aspirin in further studies.

We next investigated if aspirin could efficiently kill Fn7-1. We tested actively growing cells by subculturing Fn7-1 into sTSB or media supplemented with 5 mM or 10 mM aspirin (Fig. 1E). There was a significant decrease in CFU for both aspirin media, suggesting that aspirin was not only inhibiting growth but also reducing culture viability. In 10 mM aspirin, cell viability was reduced by ∼3 logs from the time of inoculation (t_0_). To address if aspirin could kill stationary cells, we grew Fn7-1 overnight in the absence of aspirin and then transferred cells into media containing aspirin (Fig. 1F). Under these conditions, Fn7-1 growth yield doubled only over 24 h in sTSB, supporting the conclusion that these cells were not rapidly growing and were likely in stationary phase, despite being washed into fresh medium to allow pH matching with the aspirin-supplemented media. In 5 mM aspirin, there was effectively no change in CFU over 24 h. However, when cells were washed into 10 mM aspirin, there was a 6-log decrease in Fn7-1 CFUs compared with those of t_0_, a reproducible but surprising result given the results for 5 mM. These data support that, in addition to inhibiting Fn7-1 growth, aspirin is able to reduce viable Fn7-1 cells, under both actively growing and stationary states.

### Subinhibitory concentrations of aspirin affect Fn7-1 gene expression.

We observed subtle changes in phenotypes associated with autoaggregation when Fn7-1 was grown in subinhibitory levels of aspirin. In 1 mM aspirin, cells still formed aggregates, as Fn7-1 does under these conditions, but they appeared looser (see Fig. S1A in the supplemental material). Unlike Fn7-1 grown in the sTSB where clumping leads to large fluctuations in OD_600_ during stationary phase (Fig. S1B), the OD_600_ of Fn7-1 grown in 1 mM aspirin remained more consistent (Fig. S1C). As aspirin and salicylic downregulate surface proteins in some bacteria (35, 36), we posited that aspirin may have similar effects on Fn7-1 gene expression, as suggested by these autoaggregation observations.

10.1128/mBio.00547-21.1 FIG S1 Changes to Fn7-1 autoaggregation when grown in 1 mM aspirin. (A) Fn7-1 grown in sTSB or sTSB supplemented with 1 mM aspirin demonstrates different clumping behaviors. (B and C) OD_600_ data from two individual wells of Fn7-1 grown in either sTSB or sTSB with 1 mM aspirin. For this experiment, Fn7-1 was grown in a 96-well plate to better visualize autoaggregation phenotypes. Download FIG S1, TIF file, 2.7 MB. Copyright © 2021 Brennan et al. 2021 Brennan et al. https://creativecommons.org/licenses/by/4.0/ This content is distributed under the terms of the Creative Commons Attribution 4.0 International license.

**FIG 2:**
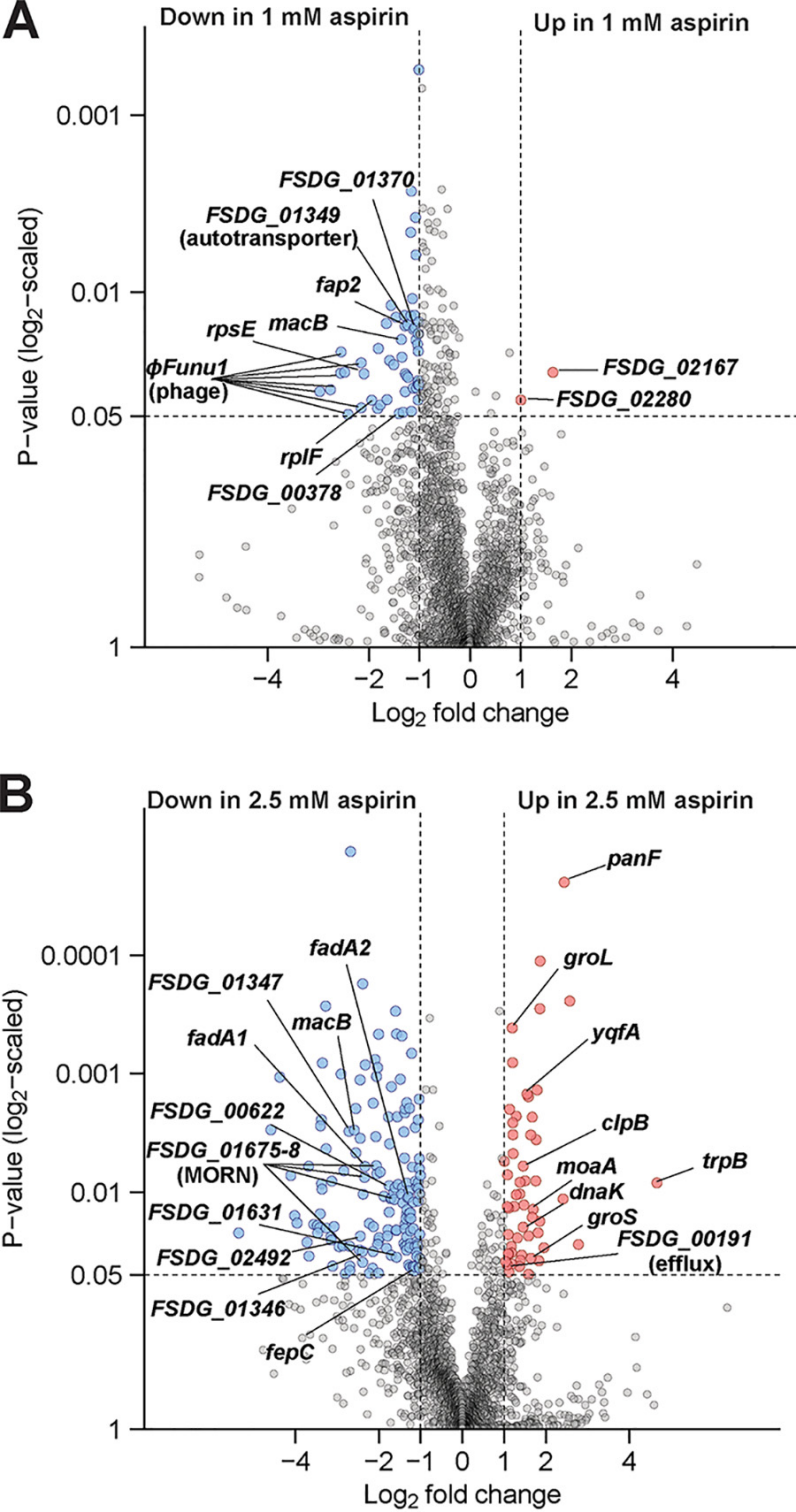
Downregulation of Fn7-1 gene expression in response to aspirin in culture. Volcano plots of RNA-seq expression data from Fn7-1 grown in sTSB compared with media supplemented with 1 mM aspirin (A) or 2.5 mM aspirin (B) for 24 h. Data represent 2 replicates for Fn7-1 grown in sTSB and 3 replicates each for Fn7-1 grown in 1 mM or 2.5 mM aspirin. Cutoffs indicate 2-fold changes in expression and *P* values <0.05 as described in the Materials and Methods. A full list of differentially expressed genes can be found in Data Set S1 and S2.

To address this possibility, we performed RNA sequencing (RNA-seq) on Fn7-1 cells grown overnight in sTSB and sTSB supplemented with 1 mM aspirin (Fig. 2A; Data Set S1 in the supplemental material). Consistent with prior studies (33, 42), we observed that this subinhibitory level of aspirin led to a largely downregulatory shift in gene expression. Using a cutoff *P* value of <0.05 and a 2-fold change, we observed 53 genes with significantly lower expression and only 2 genes (both encoding proteins of unknown function) with significantly higher expression when Fn7-1 was grown in 1 mM aspirin. Genes with reduced expression in 1 mM aspirin include *fap2*, encoding an autotransporter with important roles in CRC and other host interactions (4, 14, 43, 44), as well as *FSDG_01349* and *FSDG_01370*, also encoding autotransporters, a class of potential virulence factors abundant in F. nucleatum genomes (45). In Fn23726, the *FSDG_01349* homolog (*Gene_2067*) is also genomically linked to *fap2* (45). Another downregulated gene, *FSDG_00378*, encodes a β-barrel protein, like the autotransporters, and is similarly predicted to localize to the outer membrane. Given their subcellular localization, one of these genes may be responsible for the altered autoaggregation, but Fn7-1’s lack of genetic tractability makes it difficult to functionally test. Expression of housekeeping genes involved in transcription (*rpoC*), translation (*rpsE* and *rplF*), and cell division (*mreB*) were also reduced in response to aspirin, which may provide insight into how aspirin reduces Fn7-1 growth. Other downregulated genes include genes in the ϕFunu1 phage locus (46), a gene upregulated during Caco2 infection (*oppD5*) (47), and *macB*, encoding an efflux pump predicted to play a role in macrolide export and consistent with aspirin and salicylic acid altering antibiotic sensitivity in other bacteria (48–51). Overall, these results support a conclusion that subinhibitory aspirin alters Fn7-1 gene expression; however, our interpretation is limited by homology-based inferred functions and an overrepresentation of genes of unknown function.

10.1128/mBio.00547-21.4 DATA SET S1 Differentially expressed Fn7-1 genes in response to 1 mM aspirin. Download Data Set S1, XLSX file, 1.0 MB. Copyright © 2021 Brennan et al. 2021 Brennan et al. https://creativecommons.org/licenses/by/4.0/ This content is distributed under the terms of the Creative Commons Attribution 4.0 International license.

10.1128/mBio.00547-21.5 DATA SET S2 Differentially expressed Fn7-1 genes in response to 2.5 mM aspirin. Download Data Set S2, XLSX file, 1.0 MB. Copyright © 2021 Brennan et al. 2021 Brennan et al. https://creativecommons.org/licenses/by/4.0/ This content is distributed under the terms of the Creative Commons Attribution 4.0 International license.

To further probe how aspirin inhibits Fn7-1 growth, we assessed gene expression in 2.5 mM aspirin, a dosage at which Fn7-1 growth was reduced (Fig. 1A) while providing sufficient RNA yield. In response to 2.5 mM aspirin, 55 genes were significantly upregulated and 155 genes significantly downregulated using the same cutoff (Fig. 2B; see Data Set S2 in the supplemental material). These changes represent over 8% of the predicted coding sequences in Fn7-1, suggesting that global changes in response to this higher dosage are indicative of a general stress response. Consistent with this hypothesis, we observed upregulation of several chaperone-related genes, including *groS*, *groL*, *clpB*, and *dnaK*. Of the genes downregulated in 1 mM aspirin, 15 are also downregulated in 2.5 mM and may represent an aspirin-specific response as opposed to a general stress or death response. These genes include *macB* and its neighboring genes *FSDG_01346* and *FSDG_01347*, as well as *fepC*, predicted to be involved in iron transport. In contrast to the downregulation of *macB*, another gene predicted to encode an efflux pump protein, *FSDG_00191*, is upregulated in 2.5 mM aspirin, which together could conversely affect how aspirin might alter antibiotic sensitivity in F. nucleatum. As with 1 mM aspirin, we observed downregulation of potential virulence factors in 2.5 mM aspirin, including two genes encoding FadA-like domains (12, 52) (*FSDG_02507* and *FSDG_02530*), multiple autotransporters (*FSDG_00622*, *FSDG_01631*, and *FSDG_02492*), and a locus of genes (*FSDG_01675-01678*) predicted to encode MORN domain-containing proteins that are overrepresented in invasive F. nucleatum strains (52). These data support that aspirin alters expression of Fn7-1 genes that have the potential to affect how Fn7-1 behaves and interacts with host cells.

### Aspirin treatment reduces Fn7-1-potentiated tumorigenesis in the *Apc^Min/+^* model.

**FIG 3:**
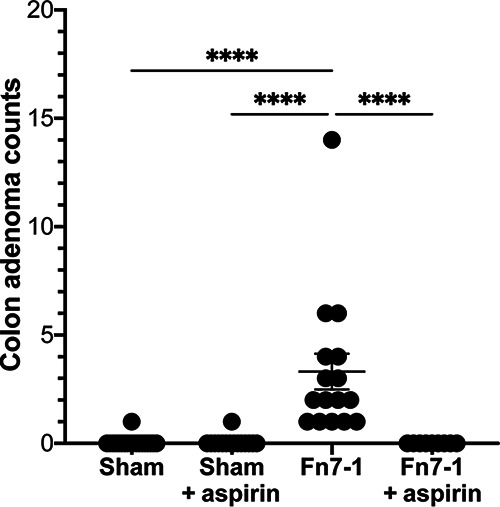
Colonic adenoma burden in the murine *Apc^Min/+^* model in response to daily Fn7-1 instillation and aspirin supplementation. Conventional specific-pathogen-free *Apc^Min/+^* mice were orally inoculated with Fn7-1 or a sham control (sTSB) daily and simultaneously maintained on either a control chow or a chow supplemented with 200 ppm aspirin, with both treatments beginning at 6 weeks of age and continuing until 14 weeks. Mice were then sacrificed, and the colons were prepared for histological analysis for enumeration of colon adenomas. Data points represent adenoma counts from individual mice, bars indicate the mean ± SEM, and statistical analysis was performed by Kruskal-Wallis test with *post hoc* Dunn’s test for multiple comparisons. ****, *P* < 0.0001.

We next sought to determine if aspirin might affect Fn7-1-potentiated intestinal tumorigenesis, given (i) the pleiotropic effects of different levels of aspirin on Fn7-1 in culture, (ii) the difficulty of determining colonic intraluminal aspirin concentration to investigate in culture-based experiments, and (iii) noted discrepancies in how bacteria respond to antimicrobial compounds *in vivo* and *in vitro* (53, 54). Using the *Apc^Min/+^* mouse model of intestinal tumorigenesis in which Fn7-1 is orally inoculated into conventionally reared mice daily beginning at 6 weeks of age (3), we concurrently transitioned mice to either a control chow or one supplemented with 200 ppm aspirin, a dosage used in previous studies that demonstrated mild, if any, effects on tumorigenesis (24, 25). After 8 weeks of treatment, consistent with our prior study (3), we observed that Fn7-1 treatment led to a significant increase in colonic adenoma burden in mice on the control chow, compared with mice orally inoculated with medium alone (sham mice) on the control chow that rarely develop colonic adenomas in our facility (Fig. 3). Similarly, sham mice placed on the aspirin chow seldomly developed colonic adenomas. In contrast, when mice treated with Fn7-1 were placed on the aspirin-supplemented chow, we saw complete abrogation of colonic adenoma development. These *in vivo* results suggest a specific role for aspirin in blocking Fn7-1-potentiated intestinal tumorigenesis.

### Comparative survey of aspirin sensitivity in F. nucleatum ATCC strains and clinical tumor isolates.

**FIG 4:**
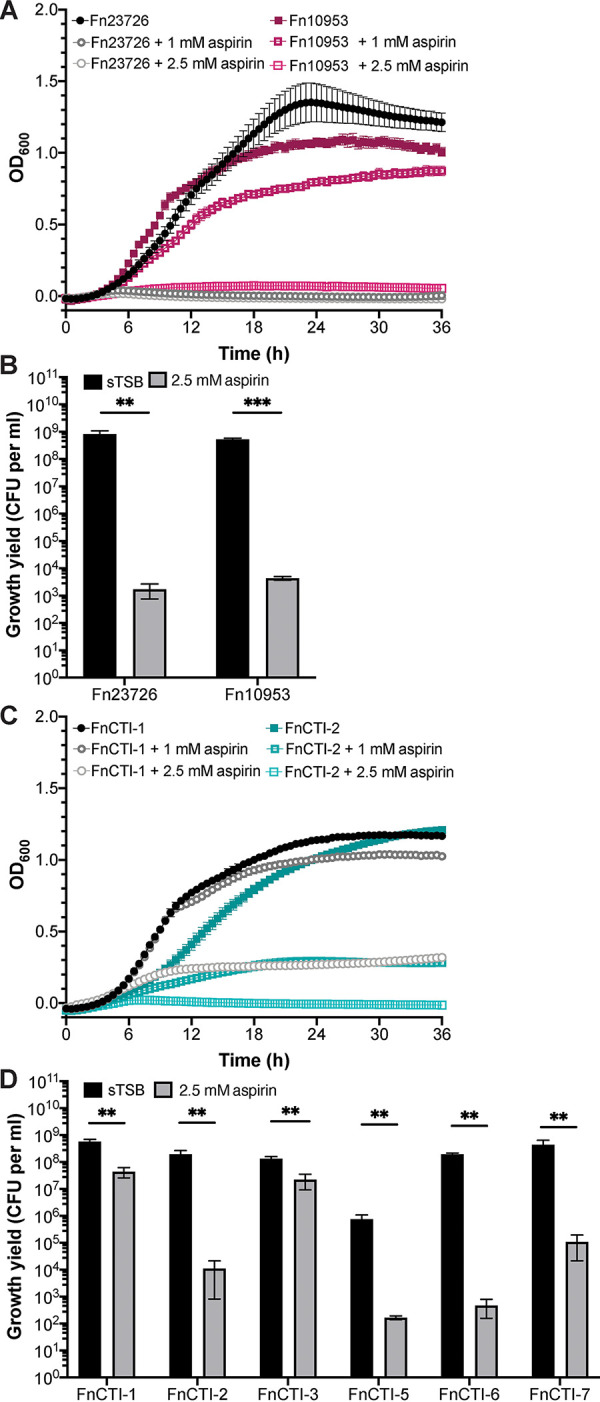
Growth inhibition of aspirin on ATCC and CRC isolates of F. nucleatum. Growth of ATCC strains Fn23726 and Fn10953 in response to aspirin as determined by optical density (A) and growth yield (CFU per ml) at 24 h (B). (C) Growth of the CRC F. nucleatum isolates FnCTI-1 and FnCTI-2 in response to aspirin as determined by optical density. (D) Growth yield of FnCTI-1, -2, -3, -5, -6, and -7 in response to 2.5 mM aspirin after 24 h as determined by CFU per ml. All data represent the mean ± SEM for at least 6 cultures. Growth curves were analyzed by two-way repeated measures ANOVA with *post hoc* Dunnett’s test. Growth was significantly different from sTSB at *P* values of <0.05 for each of the following strains at the indicated time points and concentrations: Fn23762 (1 mM and 2.5 mM at 7.5 h), Fn10953 (1 mM at 6.5 h and 2.5 mM at 5.5 h), FnCTI-1 (1 mM at 17.5 h and 2.5 mM at 7 h), and FnCTI-2 (1 mM at 9 h and 2.5 mM at 7 h). Analysis of growth yield data was performed by Mann-Whitney test. **, *P* < 0.01; ***, *P* < 0.001.

Given our results in culture and in mice, we next explored how aspirin might affect F. nucleatum as it relates to human CRC by assessing the sensitivity of other F. nucleatum strains, including isolates from human CRC tissues. We first examined how aspirin affects the ATCC strains Fn23726 (F. nucleatum subsp. *nucleatum*) and Fn10953 (F. nucleatum subsp. *polymorphum*). We performed growth curve assays for these strains in sTSB or in the presence of 1 mM and 2.5 mM aspirin (Fig. 4A). Unlike Fn7-1 which exhibited a slight growth increase at this dosage (Fig. 1A), even 1 mM aspirin was sufficient to fully inhibit Fn23726 growth. Similarly, Fn10953 demonstrated reduced growth at 1 mM and no appreciable growth at 2.5 mM aspirin. These observations were supported by strong decreases in yield (>5 logs lower CFU/ml than that in sTSB) for both Fn23276 and Fn10953 in 2.5 mM aspirin (Fig. 4B). These phenotypes demonstrated that these F. nucleatum strains are more sensitive to aspirin in culture than Fn7-1, for which we observed only ∼4-fold reduction in CFU in 2.5 mM aspirin (Fig. 1B).

Given this observation, we asked if F. nucleatum strains isolated from human CRC tissues (FnCTI-1, -2, -3, -5, -6, and -7) (4) would behave more like Fn7-1, a clinical isolate from a patient with inflammatory bowel disease (55), or like the extraintestinal and lab-adapted ATCC strains Fn23726 and Fn10953. In growth curve assays, we observed varied phenotypes. FnCTI-1 exhibited only mildly reduced growth in the presence of 1 mM aspirin and was able to grow slightly in 2.5 mM, whereas the growth of FnCTI-2 was strongly reduced in 1 mM aspirin and entirely abrogated in 2.5 mM (Fig. 4C). The greater aspirin sensitivity of FnCTI-2 was supported by larger reductions in viable CFU in the presence of 2.5 mM aspirin for FnCTI-2 (>10^4^-fold) than for FnCTI-1 (∼10-fold) (Fig. 4D). FnCTI-3 was the least aspirin-sensitive isolate, as determined by both minimal effects on growth yield (Fig. 4D) and OD_600_ (see Fig. S2A in the supplemental material). FnCTI-5, FnCTI-6, and FnCTI-7 all showed multilog decreases in growth yield by CFU in 2.5 mM aspirin (Fig. 4D) and intermediate OD_600_ phenotypes for FnCTI-6 and FnCTI-7 (Fig. S2B and C). FnCTI-5 exhibits strong clumping and cannot be accurately measured by OD_600_. Together, these data demonstrate that both CRC tumor isolates and ATCC strains of F. nucleatum are sensitive to aspirin in culture, frequently more than we observed for Fn7-1 (Fig. 1A and B), supporting the relevance of F. nucleatum aspirin sensitivity as we consider approaches to reduce fusobacterial burden.

10.1128/mBio.00547-21.2 FIG S2 Differential growth responses of F. nucleatum CTI strains in the presence of aspirin. Growth of FnCTI-3 (A), FnCTI-6 (B), and FnCTI-7 (C) in sTSB or media supplemented with 1 mM or 2.5 mM aspirin. All data represent the mean ± SEM for at least 6 cultures. Growth curves were analyzed by two-way repeated measures ANOVA with *post hoc* Dunnett’s test. Growth was significantly different from sTSB at a *P* value of <0.05 for each of the following strains beginning at the indicated timepoints and concentrations: CTI-3 (2.5 mM at 11.5 h), CTI-5 (1 mM at 8 h and 2.5 mM at 6 h), and FnCTI-7 (2.5 mM at 11 h). Download FIG S2, TIF file, 1.0 MB. Copyright © 2021 Brennan et al. 2021 Brennan et al. https://creativecommons.org/licenses/by/4.0/ This content is distributed under the terms of the Creative Commons Attribution 4.0 International license.

### Effect of aspirin on growth of CRC-associated enterotoxigenic B. fragilis and colibactin-producing E. coli.

**FIG 5:**
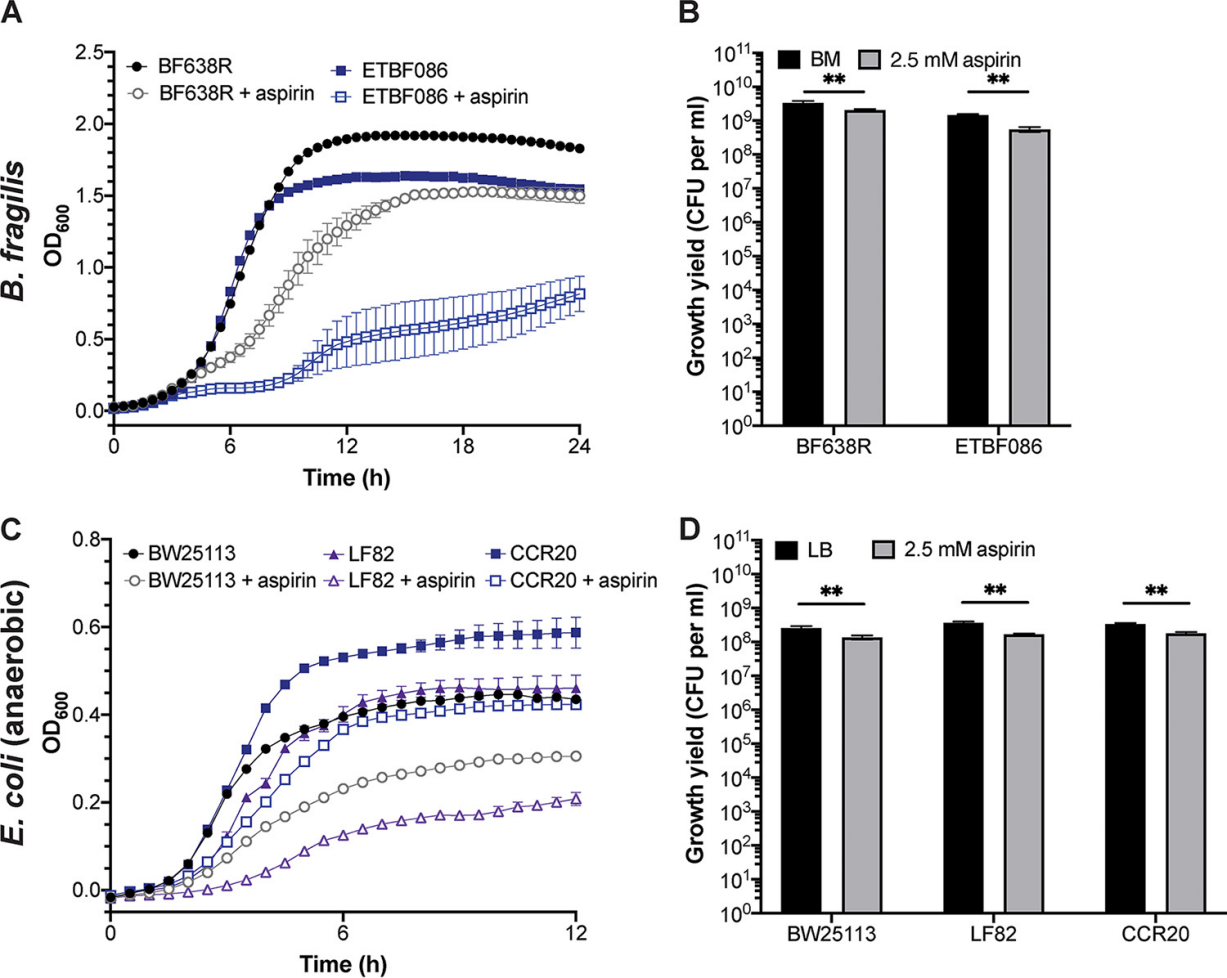
CRC-associated microbe B. fragilis and E. coli responses to aspirin. Growth of B. fragilis strains BF638R and ETBF086 in basal medium (BM) or BM supplemented with 2.5 mM aspirin as determined by optical density (A) and growth yield (CFU per ml; B) after anaerobic growth for 24 h at 37°C. Growth of E. coli strains BW25113, LF82, and CCR20 and ETBF086 in LB or LB supplemented with 2.5 mM aspirin as determined by optical density (C) and growth yield (CFU per ml; D) after anaerobic growth for 24 h at 37°C. All data represent the mean ± SEM for at least 6 cultures. Growth curves were analyzed by two-way repeated measures ANOVA with *post hoc* Dunnett’s test, and growth in 1 mM aspirin was significantly different from control medium at *P* values of <0.05 for each of the following strains beginning at the indicated time points: BF638R (6 h), ETBF086 (5.5 h), BW25113 (3 h), LF82 (3.5 h), and CCR20 (3.5 h). Growth yield analysis was performed by Mann-Whitney test; **, *P* < 0.01.

Beyond F. nucleatum, two of the best-characterized CRC-associated microbes are enterotoxigenic B. fragilis (ETBF), which drives a strong proinflammatory and protumorigenic response, and colibactin-producing E. coli, which induces DNA damage and a specific mutational signature that can be found in CRC tissues and increases tumor multiplicity in preclinical mouse models (1, 2, 5, 56–59). We used growth assays on ETBF and colibactin-producing E. coli, as well as additional B. fragilis and E. coli strains, to ascertain if these CRC-associated bacteria are similarly sensitive to aspirin and could represent additional targets for aspirin modulation. For B. fragilis, we investigated ETBF086, an enterotoxigenic isolate, and BF638R, a nontoxigenic isolate. Both B. fragilis strains exhibited reduced growth in 2.5 mM aspirin (Fig. 5A and B). However, while statistically significant, the effects were far milder than we observed for many of the F. nucleatum strains, and represented only less than 3-fold decreases in growth yield.

For E. coli, we investigated the following: BW25113, a K-12 strain; LF82, an adherent-invasive strain; and CCR20, a colibactin-producing strain isolated from CRC tissues. All strains exhibited reduced growth in 2.5 mM aspirin compared with that in Luria-Bertani (LB) (Fig. 5C and D). Much like B. fragilis, however, the effects were statistically significant but mild, reducing growth yield by ∼2-fold. Because we performed the growth assays under anaerobic conditions, even for E. coli, we also determined growth yield for the E. coli strains in 2.5 mM aspirin under aerobic conditions and again observed minimal aspirin sensitivity (see Fig. S3 in the supplemental material). Taken together, these data suggest that, although both B. fragilis and E. coli exhibited slight growth inhibition in response to aspirin, many F. nucleatum strains are far more aspirin sensitive, at least under the conditions of our assays. Therefore, the observations we make using our mouse models and human study regarding the effects of aspirin on F. nucleatum *in vivo* may not extend to these other CRC-associated microbes.

10.1128/mBio.00547-21.3 FIG S3 Aspirin minimally affects E. coli strains grown under aerobic conditions. Growth yield (CFU per ml) of E. coli strains BW25113, LF82, and CCR20 in LB or LB supplemented with 2.5 mM aspirin for 18 h at 37°C under aerobic conditions. All data represent the mean ± SEM for at least 6 cultures. Growth yield analysis was performed by Mann-Whitney test and ** indicates a *P* value of <0.01. Download FIG S3, TIF file, 0.3 MB. Copyright © 2021 Brennan et al. 2021 Brennan et al. https://creativecommons.org/licenses/by/4.0/ This content is distributed under the terms of the Creative Commons Attribution 4.0 International license.

### Fusobacterial abundance is reduced in colonic adenomas from patients who use aspirin.

**FIG 6:**
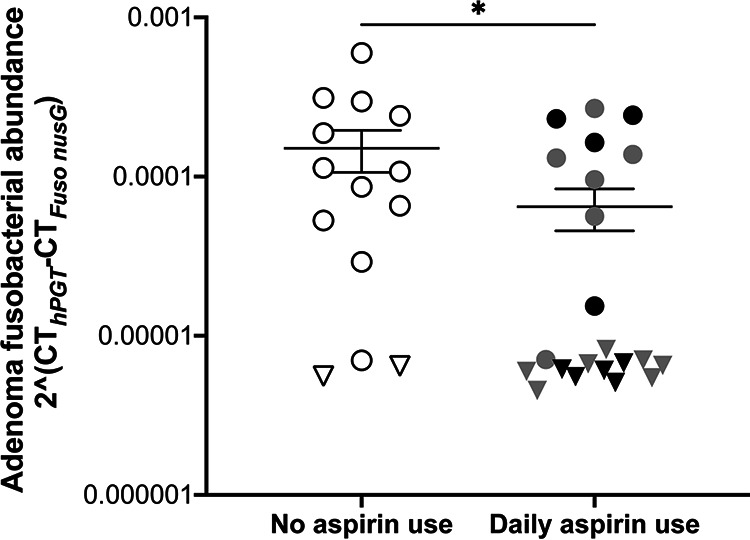
Fusobacterial abundance in human colonic adenomas of patients who self-reported aspirin use. qPCR was performed on DNA isolated from human colonic adenomas using primers targeting the *nusG* gene of *Fusobacterium* spp. and the human *PGT* gene to normalize for human DNA abundance. Samples were grouped based on patients who reported daily aspirin use and those who reported no aspirin use (white data points). For patients reporting daily aspirin use, gray data points indicate low-dose aspirin (<325 mg daily) and black data points indicate those reporting high-dose aspirin (≥325 mg daily). Data points (circles) represent individual adenomas, and triangles represent adenomas for which *Fusobacterium* sp. abundance was below the limit of detection and marked at the limit of detection based on the cycle threshold (CT) for *hPGT* in that individual sample. Bars indicate the mean ± SEM. Analysis was performed by Mann-Whitney test, with * indicating *P* values of <0.05.

Given the aspirin sensitivity of F. nucleatum, including isolates from human CRC tissues, that we observed in culture, we posited that regular aspirin use may affect fusobacterial load in human tissues. As F. nucleatum is infrequently detected in healthy human stool samples, we used human colonic adenoma tissues, in which F. nucleatum is enriched (3, 10, 11), to determine if aspirin affects fusobacterial burden. We isolated DNA from 36 human adenoma samples and determined *Fusobacterium* sp. abundance, normalized to human *PGT* gene copies as a reference for adenoma DNA abundance, by quantitative PCR (qPCR) as previously described (8, 16). After data analysis when we were no longer blind to aspirin use, we compared the relative adenoma fusobacterial abundance across patients who reported taking aspirin daily and those whose reported no aspirin use (Fig. 6). We observed a 2.3-fold lower mean fusobacterial burden for patients taking aspirin. However, if we instead categorized adenomas as *Fusobacterium* high or *Fusobacterium* low/negative, as often done in the epidemiological literature, we observed 7 of 14 adenomas as *Fusobacterium* high in the control group and only 6 of 22 adenomas as *Fusobacterium* high in the aspirin group, using a relative fusobacterial load cutoff of 0.0001. We further analyzed the data comparing patients reporting low-dose (<325 mg daily) or high-dose (≥325 mg daily) aspirin use and found no dose-related differences between the two groups (Mann-Whitney test, *P* > 0.9999). These data demonstrate an association between aspirin use and reduced *Fusobacterium* sp. load in precancerous adenomas and suggest that the aspirin modulation of F. nucleatum we have described in culture may also occur in humans. Unfortunately, we were unable to determine the carriage of ETBF or colibactin-producing E. coli in the human adenoma samples we examined, as growth to enrich for either B. fragilis or E. coli isolates is required to assess their presence (58, 60) rather than direct lysis of the tissue, as performed for *Fusobacterium* sp. quantification. Thus, we could not test if these bacteria are less affected by aspirin use as our *in vitro* data might suggest.

## DISCUSSION

In this work, we have coupled complementary culture-based experiments, a murine model of intestinal tumorigenesis, and human tissues to reveal that aspirin, a CRC chemopreventive drug, has antibacterial activity against F. nucleatum. More broadly, the effects of aspirin on F. nucleatum we observe demonstrate the merit of reconsidering existing preventative and therapeutic options and investigating how they may also be affecting the CRC-associated microbiome beyond their more well-known mechanisms.

In culture, we demonstrated the sensitivity of F. nucleatum strains and other CRC-associated bacteria to millimolar levels of aspirin. Although relatively high compared with more canonical antimicrobial compounds, these levels of aspirin are comparable to those used to study the effect of aspirin on other bacteria and colon cancer cell lines and those observed in human serum in some studies, supporting their relevance (31, 41, 61–63). At these levels, all F. nucleatum strains demonstrated aspirin sensitivity. The responses of Fn7-1 to aspirin at these doses were often surprisingly stark, such as a 6-log reduction in stationary Fn7-1 CFU in 10 mM aspirin compared with no reduction in 5 mM aspirin. Similarly, the range of aspirin levels used in our study often covered the inflection point for sensitivity of other tested strains, which may partially explain why we were able to observe differences across F. nucleatum strains. The underlying basis for why some F. nucleatum strains exhibit stronger aspirin sensitivity may inform the method of action for how aspirin affects F. nucleatum and help explain the results we observed for Fn7-1. An experimental evolution study of benzoate tolerance in E. coli observed concomitant salicylic acid resistance in the benzoate-adapted strains, whose mutations largely mapped to multidrug efflux systems (64). Variation in either the presence or expression of these systems, such as the MacB macrolide transporter whose expression in Fn7-1 was downregulated in response to aspirin, across F. nucleatum strains may be one explanation for different aspirin sensitivity. Isolation and characterization of F. nucleatum mutants that exhibit improved growth directly in aspirin or salicylic acid, while beyond the scope of this project, might reveal specific mechanisms underlying fusobacterial aspirin sensitivity and resistance. In addition to directly inhibiting bacterial growth, aspirin can alter sensitivity to antibiotics (48–51), which could synergize to further reduce F. nucleatum levels *in vivo*, although it is yet to be empirically tested in F. nucleatum.

Beyond culture-based experiments, we investigated how aspirin influences F. nucleatum in the setting of CRC using a genetically driven mouse model to show that aspirin abrogated F. nucleatum-potentiated tumorigenesis. Experimental limitations of this model—including the lack of colonic adenomas in either the absence of Fn7-1 or in Fn7-1 mice on aspirin chow and the difficulty in isolating viable Fn7-1 from the stool of conventional (specific pathogen free) mice—left us unable to fully ascertain if this observation was due to aspirin directly altering Fn7-1 viability or behavior or dominant effects of aspirin on inflammation and colonic tumor development independent of Fn7-1. However, aspirin leads to only mild, if any, effects on spontaneous intestinal adenoma formation in *Apc^Min/+^* mice at this dosage (24, 25). Furthermore, a recent study proposed a role for the microbiota in mediating the response to aspirin in this model, as intestinal tumor burdens were reduced only when the microbiota was also disrupted by antibiotics (27). Taken together, these studies suggest that it is unlikely that the anti-inflammatory effects of aspirin alone are sufficient for the strong inhibition of Fn7-1-potentiated tumorigenesis we observed, and therefore, there likely may be a role for aspirin modulation of Fn7-1 in this phenotype. F. nucleatum aspirin-resistant mutants, as proposed earlier, would also prove useful in more definitively probing the role of aspirin-sensitivity in blocking F. nucleatum-potentiating tumorigenesis in the *Apc^Min/+^* model, representing a potential future direction for *in vivo* experimentation. Beyond aspirin sensitivity, our observations could also be influenced by changes in Fn7-1 gene expression in response to lower levels of aspirin, including downregulation of important virulence factors like *fap2*, required to mediate CRC tissue localization and anti-tumor immunity (4, 14), and *fadA*, encoding a fusobacterial adhesin that engages E-cadherin to promote Wnt/β‐catenin signaling and drive cancer cell proliferation (12).

Using human samples, we demonstrated reduced fusobacterial load in colonic adenomas for individuals who self-reported daily aspirin use, by both mean fusobacterial burden and categorization as *Fusobacterium* high or *Fusobacterium* low/negative. The poorer prognosis and development of chemoresistance associated with fusobacterial load were determined using analyses with stratification of *Fusobacterium*-high and *Fusobacterium*-low/negative CRC tissues (15, 17) rather than based on correlation to a continuum of fusobacterial load. Therefore, the categorical threshold differences in fusobacterial load in response to aspirin use may indicate a greater potential to shape CRC outcomes than the 2.3-fold difference in mean adenoma fusobacterial abundance may suggest. Important remaining questions include where in the body (e.g., the oral cavity, the intestinal tract, or within the tumor microenvironment) and when (relative to tumor development) F. nucleatum exposure is occurring so that aspirin can be used most effectively.

The effect of aspirin on F. nucleatum growth and behavior is only one aspect of the interactions between aspirin and the microbiota in the context of colorectal tumorigenesis. For example, we previously demonstrated that F. nucleatum drives expression of *Ptgs2* (3), which encodes COX-2, the predominant cellular target of aspirin in its anti-inflammatory role. Furthermore, a recent study suggested a role of the microbiota in influencing aspirin bioavailability in intestinal tumor models (27). Thus, the interactions among aspirin, F. nucleatum, other microbiota constituents, and inflammation in CRC tissues likely represent a highly complex interplay rather than the reductionist focus here. The critical question is how we use our understanding of these interactions to inform personalized microbiota-based medicine, such as more strongly recommending aspirin to individuals who harbor detectable fecal F. nucleatum and therefore are at a higher risk for CRC in a microbiota-based screening (65, 66).

## MATERIALS AND METHODS

### Bacterial strains and growth conditions.

F. nucleatum strains Fn7-1 (55); FnCTI-1, -2, -3, -5, -6, and -7 (4); Fn23726; and Fn10953 (ATCC; Manassas, VA) were grown in Columbia broth or tryptic soy broth supplemented with hemin (5 μg/ml) and menadione (1 μg/ml) (sTSB) at 37°C, under anaerobic conditions using a vinyl chamber (Coy Lab Products, Grass Lake, MI). Fastidious anaerobe agar (FAA; Neogen, Lansing, MI) supplemented with 5% defibrinated sheep blood was used for plating. E. coli strains BW25113 (67), LF82 (68), and CCR20 (59, 69) were grown in Luria-Bertani (LB) broth at 37°C under aerobic or anaerobic conditions, as indicated, and plated onto either LB or MacConkey agar. B. fragilis strains BF638R (70) and ETBF 086-54443-2-2 (ETBF086) (71) were grown in supplemented basal medium (BM; 20 g/liter proteose peptone, 5 g/liter yeast extract, 5 g/liter sodium chloride, 5 g/liter glucose, 5 g/liter dipotassium phosphate, 0.5 g/liter l-cysteine, 5 μg/ml hemin, 2.5 μg/ml vitamin K1; modified from reference 72) at 37°C under anaerobic conditions and plated onto FAA supplemented with 5% defibrinated sheep blood.

All media supplemented with aspirin or salicylic acid were pH adjusted to match the control medium. For growth experiments, bacteria were grown overnight and then subcultured 1:100 into indicated media for 24 h at 37°C, unless otherwise noted. OD_600_ was measured in 48-well plates, unless otherwise indicated, using a Biotek Eon plate reader (Winooski, VT), located within the anaerobic chamber.

### Gene expression studies.

Fn7-1 was grown for 24 h under the conditions described. RNA was extracted from the cultures by using a Directzol RNA Miniprep kit (Zymo Research, Irvine, CA), followed by Turbo DNA-free treatment (Invitrogen, Carlsbad, CA) and concentration with an RNeasy MinElute cleanup kit (Qiagen, Germantown, MD). RNA quality assessment (2200 Tapestation; Agilent, Santa Clara, CA), rRNA depletion (Ribo-Zero rRNA removal kit for bacteria; Illumina, San Diego, CA), library construction (Wafergen PrepX directional RNA-seq library kit; TaKaRa Bio, Mountain View, CA), and sequencing (HiSeq 2500; Illumina) were performed by the Harvard Medical School (HMS) Biopolymers Core Facility using standard techniques to generate 75-base pair paired-end reads.

Raw sequencing reads were quality trimmed using Trimmomatic (73) (v0.39) configured to perform sliding window scan with the following settings: “ILLUMINACLIP:${adapter_library_FASTA}:2:36:7:1: keepBothReads LEADING:3 TRAILING:3 SLIDINGWINDOW:4:15 MINLEN:36.” Optical and PCR duplicate sequences were then identified and removed using Clumpify with the option “dedupe optical spany adjacent subs = 0” from the BBTools bioinformatic suite (http://sourceforge.net/projects/bbmap). We indexed 2,419 Fn7-1 genomic loci identified by the GenBank database (assembly accession GCA_000158275.2) using Burrows-Wheeler Aligner (BWA; v0.7.17) (74) to create a gene database for short read alignment. Low-complexity gene subsequences were hard masked with the DUST program (75) prior to read mapping with the maximal exact match (mem) algorithm and default penalty scoring strategy (76). To maximize the biological interpretation of gene expression data, we used Prokka (77) to reannotate genome loci with protein families by incorporating profile hidden Markov model (pHMM) databases from Pfam (78) (prioritized; v33), EggNOG bacteria archaea viruses (79) (v5), KOfam (80) (v96), TIGRFAMs (81) (v15), and PSORTb (82) (v3.0.2) to predict subcellular localization of proteins in Gram-negative bacteria. A gene expression matrix was constructed by counting only reads that mapped to a single gene (i.e., unambiguous reads) using the htseq-count script included with the HTSeq python library (83). Read counts were normalized to transcripts per kilobase million (TPM) to equalize variable sequencing depth across samples for differential gene expression analysis by least square linear regression as implemented in the limma R/Bioconductor package (84). Fold change between treatment conditions for each gene was calculated by determining the mean value of all combinatorial pairs of samples that fell within the interquartile range (85). To ensure robust statistical analysis, we aligned reads against a fusobacterial clade-specific marker gene collection using the ChocoPhlAn pangenome database (v30) (86).

### Animal studies.

Beginning at 6 weeks of age and continuing daily for 8 weeks, male and female *Apc^Min/+^* (sourced from Jackson and bred in-house in a barrier facility) mice were orally instilled with either 10^8^ CFU of Fn7-1 (in <100-μl volume) or medium control (sTSB). Concurrently, animals were placed on either an AIN-76A diet or AIN-76A supplemented with 200 ppm aspirin (Research Diets, Inc., New Brunswick, NJ). At 14 weeks, mice were euthanized and colons were excised for histological analysis conducted in a blind manner by J.N.G., as previously described (3). All experiments were approved and carried out in accordance with HMS’s Standing Committee on Animals and the National Institutes of Health guidelines for animal use and care.

### Determination of *Fusobacterium* sp. abundance in human colon adenomas.

Human colonic adenomas were acquired from the Pitt Biospecimen Core (PBC) at the University of Pittsburgh. Acquisition of the samples was institutional review board (IRB) approved, and informed consent was received from all participants, who also completed questions about NSAID and aspirin use. All adenomas were ≥1 cm in size. Upon endoscopic removal, they were placed into a saline ice bath and transported to pathology for sectioning. The pathologist allocated tissue for clinical diagnosis and research purposes, which was flash frozen and stored at −80°C.

DNA was isolated from adenoma tissues (∼2 mm by 2 mm by 2 mm) by overnight lysis (100 mM Tris-HCl [pH 8.5], 5 mM EDTA [pH 8.0], 0.2% sodium dodecyl sulfate, 200 mM NaCl, and 1 mg/ml proteinase K; rotating at 55°C), followed by a standard phenol-chloroform extraction. qPCR was performed on tumoral DNA (160 ng per reaction, technical duplicates) using the Kapa ProbeFast Rox low kit (Wilmington, MA) on an Agilent Mx3005P cycler. Primers targeted *Fusobacterium* spp. (forward, 5′-CAACCATTACTTTAACTCTACCATGTTCA-3′; reverse, 5′-GTTGACTTTACAGAAGGAGATTATGTAAAAATC-3′; probe, 5′-6-6-carboxyfluorescein [FAM]-TCAGCAACTTGTCCTTCTTGATCTTTAAATGAACC-black hole quencher [BHQ]1-3′) and human *PGT* (forward, 5′-ATCCCCAAAGCACCTGGTTT-3′; reverse, 5′-AGAGGCCAAGATAGTCCTGGTAA-3′; probe, 5′-6-FAM-CCATCCATGTCCTCATCTC-BHQ1-3′) as previously described (8, 16). We were blind to aspirin use until after data analysis.

### Statistical analysis.

Graphs and statistical analysis were generated using Prism 9 (GraphPad Software, San Diego, CA). Statistical tests used for each analysis are described in figure legends.

### Data availability.

RNA-seq data used in this study have been deposited in the NCBI SRA database under the BioProject identifier (ID) PRJNA701284.
