# Supplementary figures and images for: Aspirin Modulation of the Colorectal Cancer-Associated Microbe Fusobacterium nucleatum
Source: mBio. 2021 Apr 6;12(2):e00547-21. doi: 10.1128/mBio.00547-21 (PMC8092249; doi:10.1128/mBio.00547-21)

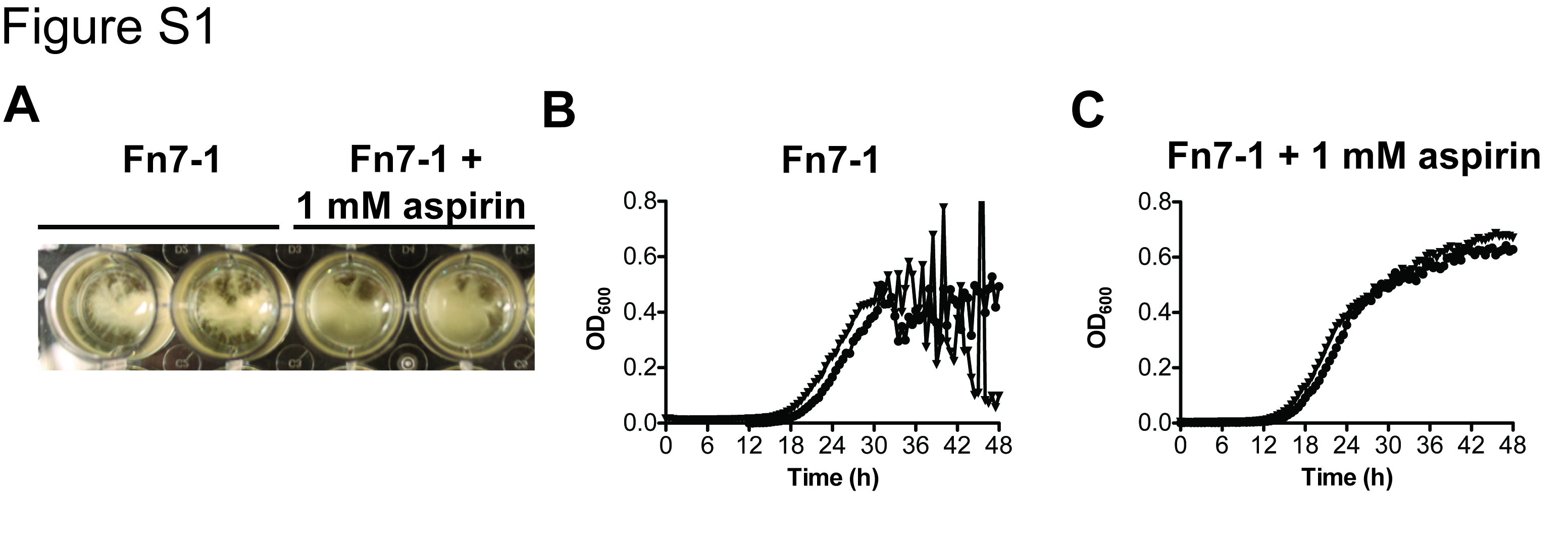

Supplement: FIG S1 [file mBio.00547-21-sf001.tif]

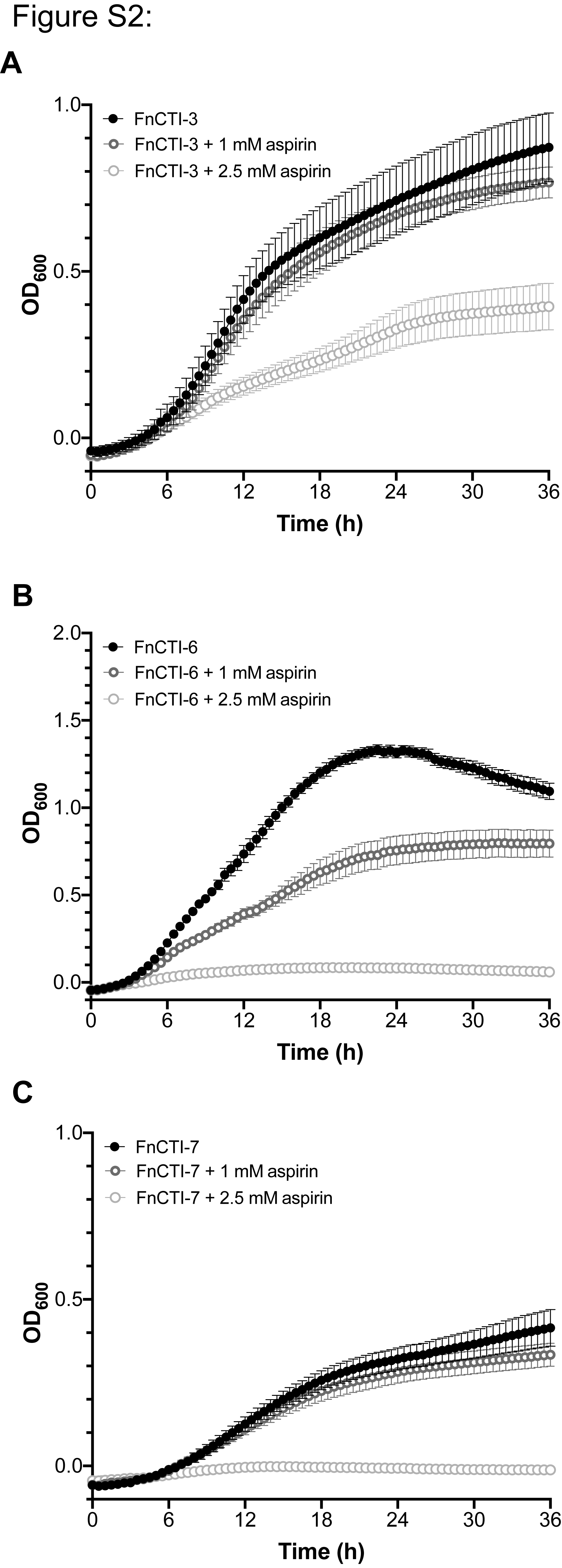

Supplement: FIG S2 [file mBio.00547-21-sf002.tif]

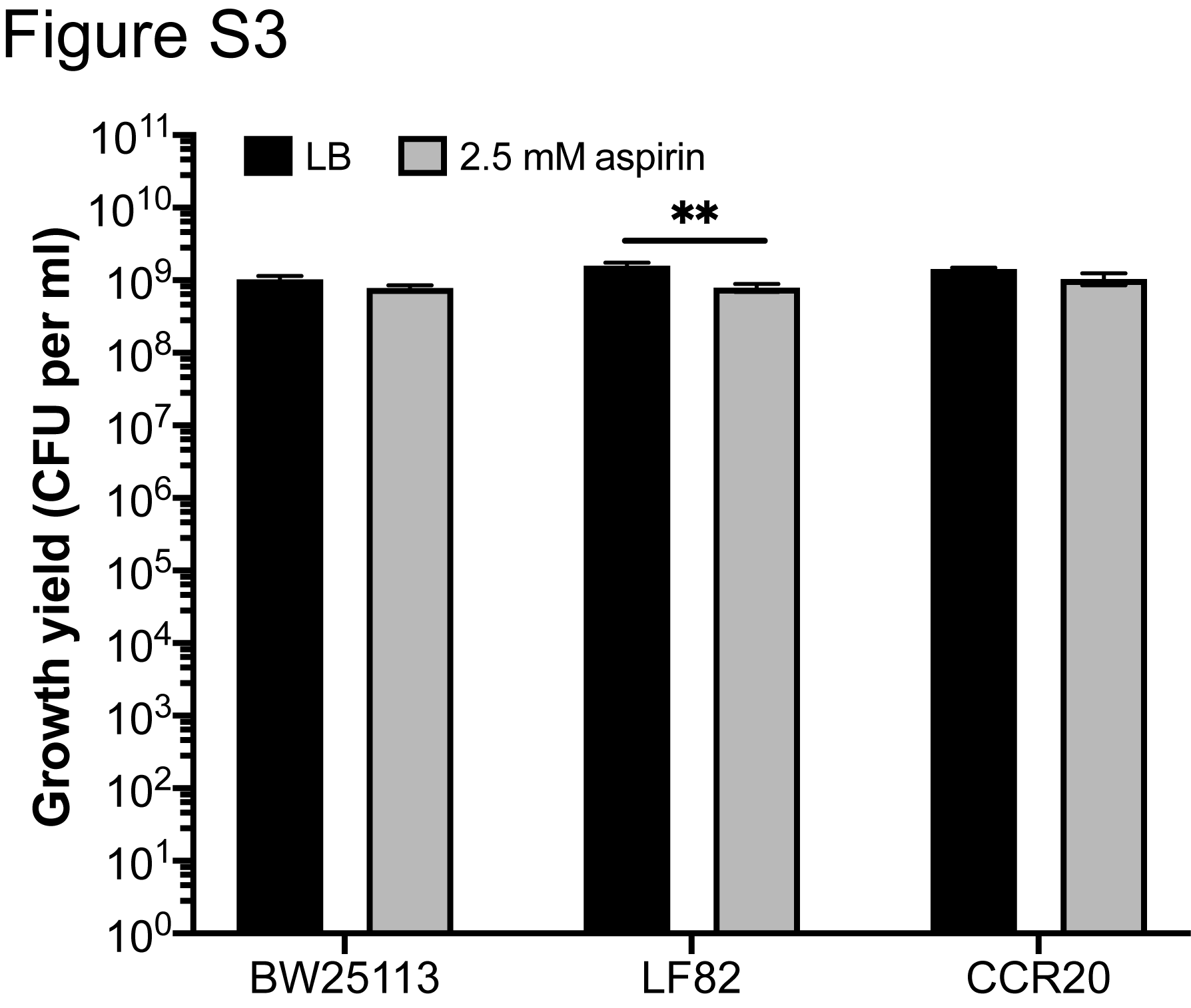

Supplement: FIG S3 [file mBio.00547-21-sf003.tif]
